# How Anticipated Emotions Guide Self-Control Judgments

**DOI:** 10.3389/fpsyg.2019.01614

**Published:** 2019-07-23

**Authors:** Hiroki P. Kotabe, Francesca Righetti, Wilhelm Hofmann

**Affiliations:** ^1^Center for Decision Research, University of Chicago Booth School of Business, Chicago, IL, United States; ^2^Department of Social and Organizational Psychology, Vrije Universiteit Amsterdam, Amsterdam, Netherlands; ^3^Department of Psychology, Ruhr University Bochum, Bochum, Germany

**Keywords:** anticipated emotions, affective forecasting, mixed emotions, self-control, self-regulation

## Abstract

When considering whether to enact or not to enact a tempting option, people often anticipate how their choices will make them feel, typically resulting in a “mixed bag” of conflicting emotions. Building on earlier work, we propose an integrative theoretical model of this judgment process and empirically test its main propositions using a novel procedure to capture and integrate both the intensity and duration of anticipated emotions. We identify and theoretically integrate four highly relevant key emotions, pleasure, frustration, guilt, and pride. Whereas the former two (basic hedonic) emotions are anticipated to dissipate relatively quickly, the latter two (self-conscious) emotions are anticipated to be more long-lived. Regarding the relative weighting of emotions, we obtained evidence for a relative guilt bias and pride neglect under default conditions. Furthermore, we identify situational influences on this judgment process and find that rendering self-conscious emotions more situationally salient positively impacts self-control decision-making. We discuss how these findings build on an integrative theory of self-control and how they are useful for the design of choice environments and interventions.

## Introduction

People’s choices are often guided by the feelings they anticipate. In a simple situation, the anticipation of positive affect may guide approach whereas the anticipation of negative affect may guide avoidance. In situations involving self-control dilemmas, however, choices present people with possible futures involving mixed and conflicting emotional experiences (Hofmann et al., 2013). For example, a dieter may anticipate that the experience of eating a medium-rare steak for dinner will involve some positive affect but the experience of breaking their diet will involve some negative affect. On the other hand, this hungry dieter may anticipate that the experience of forgoing the medium-rare steak will involve some negative affect but the experience of sticking to their diet will involve some positive affect. What can be seen in this example is that self-control does not only involve a dilemma between “being good” or “being bad,” but it also can involve the anticipation of mixed-emotional dilemmas of the possibility of feeling good *and* bad from being good *or* bad (Giner-Sorolla, 2001; Berrios et al., 2015).

What is common in such reflective choice situations is that the hypothetical scenarios associated with each choice option are imagined and simulated (Mellers and McGraw, 2001). For example, the dieter making dinner plans imagines eating a medium-rare steak vs. eating a cobb salad and then imagines what life would be like with one outcome or another. These possible futures, based on learning experience, are associated with affective responses. These forecasted affective responses, in turn, guide judgments about what to do – for example, whether or not to enact self-control when making the choice.

In this article, we propose a model of how anticipated emotions guide self-control judgments in reflective self-control situations and we report four studies testing its main propositions by addressing the following interrelated questions: (1) What are the primary emotions anticipated? (2) How do the anticipated intensities and durations of these emotions compare with each other? (3) To what degree are each of these anticipated emotions weighted into self-control judgments? Furthermore, we address questions regarding how contextual factors influence the process, including (4) the effects of increasing the situational salience of *basic hedonic emotions* (i.e., emotions that are ancient, relatively simple, and do not require self-awareness) vs. *self-conscious emotions* (i.e., emotions that are phylogenetically younger, relatively complex, reflect a self-evaluation, and do require self-awareness) involved in self-control?

### Anticipated Emotions and Decision-Making

There is considerable evidence that anticipated emotions guide human decisions, intentions and behavior. For instance, a highly influential theory of how people make behavioral intentions, the theory of planned behavior, proposes that there are three major determinants of behavioral intentions – attitude toward the behavior, subjective norms, and perceived behavior control (Ajzen, 1991). More recent research suggests that anticipated emotions are an additional major determinant (Perugini and Bagozzi, 2001). Several streams of research corroborate this claim. In the domain of risk-taking decisions, people have been shown to base risky choices not only on utilities but also on subjective calculations that take into account anticipated pleasure and the likelihood of experiencing that pleasure (Mellers, 2000; Mellers and McGraw, 2001). In the domain of purchasing decisions, people who anticipate regret when purchasing are more likely to purchase conventional or well-known options because they anticipate regretting these decisions less (Simonson, 1992). Moreover, anticipating satisfaction when making a purchase increases vivid imagery of purchase options, which results in increased weighting of vivid attributes into purchasing decisions (Shiv and Huber, 2000).

Germane to the present study, researchers have also investigated anticipated emotions in specific self-control contexts (Klass, 1990; Bagozzi and Pieters, 1998; Richard et al., 1998; Mellers, 2000; Perugini and Bagozzi, 2001; Bagozzi et al., 2003; Idson et al., 2004). For instance, anticipated positive emotions associated with goal achievement predict intentions to diet and exercise (Perugini and Bagozzi, 2001), and anticipated negative emotions associated with goal failure predict intentions to achieve idiosyncratic self-control goals (Bagozzi et al., 2003). Furthermore, anticipated self-conscious emotions including shame and guilt predict increased condom use intentions and behaviors among undergraduates (Hynie et al., 2006). Although these studies corroborate that anticipated emotions guide self-control judgments, they do not provide an integrative theoretical model for how multiple anticipated emotions are involved in the self-control judgment process.

There is at least one such model by MacInnis and Patrick (2006). They proposed that anticipated pleasure from temptation enactment and anticipated deprivation/regret from temptation nonenactment increase impulsive behavior whereas anticipated pride from temptation nonenactment and anticipated guilt/shame from temptation enactment increase impulse control. These anticipated emotions result in various motivational conflicts based on their approach or avoidance orientation. A subsequent empirical study (Patrick et al., 2009) provided empirical evidence supporting the proposed role of anticipated pride, and provided evidence that anticipating pride facilitates self-control.

In the model we set forth, we expand on their model and provide the first empirical test of several new hypotheses derived from our expanded model. Our model is not meant to replace theirs, but rather to expand on the topic by focusing on different aspects of the process, and in different circumstances. Their model specifies which anticipated emotions motivate self-control or impulse-control but does not make general predictions regarding which emotions will be weighted more or less into self-control judgments, though they highlight this topic as a future direction to which we respond here. Moreover, taking a more fine-grained approach, we integrate both anticipated emotion intensity and duration into an “area-under-the-curve” model of anticipated affect (see Study 2). This allows us to better understand how the basic hedonic vs. self-conscious nature of a given emotion affects its anticipated time course, and how people may combine intensity and duration information into their judgment.

## A Model of Anticipated Emotions in Self-Control (Maesc)

In our theoretical development, our overarching goal was to provide a general analysis of how mixed and conflicting anticipated emotions guide self-control judgments. There are numerous emotions implicated in self-control (Baumeister et al., 1994). Our intention was not to model how every one of these emotions is anticipated and weighted into self-control judgments. Rather, we focused on identifying core “self-control emotions” and modeling their role in the self-control judgment process. In the following section, we elaborate on the proposed self-control emotions, how they conceptually relate with one another, and how their anticipation guides self-control judgments.

**FIGURE 1:**
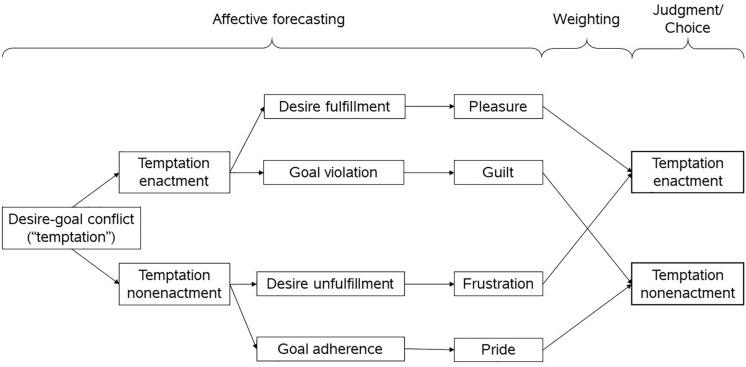
The anticipated emotions in self-control (MAESC) model. The recognition of desire-goal conflict initiates a process leading to a judgment favoring more or less self-control. Mixed and conflicting anticipated emotions mediate this process. Temptation enactment has a positive aspect – desire fulfillment – and a negative aspect – goal violation. The former is mainly associated with anticipated pleasure whereas the latter is mainly associated with anticipated guilt. Temptation nonenactment also has a positive aspect – goal adherence – and a negative aspect – desire unfulfillment. The former is mainly associated with anticipated pride whereas the latter is mainly associated with anticipated frustration. These anticipated emotions are differentially weighted into self-control judgments depending on situation and person factors.

We align MAESC within the general framework of integrative self-control theory (Kotabe and Hofmann, 2015). In this theoretical framework, a response conflict between a desire and a higher-order goal triggers effortful control processes, which involve both the motivation and the capacity to control desire. The output of the interplay of both – control effort – competes with desire strength to determine a prevailing “psychological force” (Lewin, 1951; see also Hoch and Loewenstein, 1991). The psychological force determines the behavioral outcome, given that it overcomes the enactment constraints working against it. We assume that the anticipation of mixed emotions occurs as part of a judgment process triggered by the recognition of a desire-goal conflict. Anticipated emotions are assigned weights which guide judgments favoring either less or more self-control. This process can be represented as a decision tree which forms the basis of MAESC: As shown in Figure 1, following the recognition of desire-goal conflict, one mentally simulates enacting or not enacting desire and anticipates the emotions experienced as a result of their choice (Mellers and McGraw, 2001). In simulating desire enactment, one may consider a positive aspect, *desire fulfillment*, or a negative aspect, *goal violation*. As for simulating desire nonenactment, one may consider a positive aspect, *goal adherence*, or a negative aspect, *desire unfulfillment*. We propose that each of these possible futures is associated with the anticipation of a core self-control emotion as follows: (a) desire → fulfillment pleasure, (b) goal → violation guilt, (c) goal adherence → pride, and (d) desire unfulfillment → frustration.

### Enacting Temptation: The Pleasure of Desire Fulfillment and the Guilt of Goal Violation

People widely acknowledge the role of both pleasure and guilt in self-control dilemmas, as evident in the phrase, “guilty pleasures.” From a historical perspective, pleasure and guilt are central to psychoanalytic theory (Freud, 1920), with the primeval and animalistic id driven by the “pleasure principle” and failure to live up to the moralistic superego punishable by guilt. Recently, researchers have corroborated that pleasure and guilt are (anticipated) emotional consequences of temptation enactment (MacInnis and Patrick, 2006; Hofmann et al., 2013). Guilt is also extensively discussed by Tangney and Dearing (2002) in their book “Shame and Guilt,” in which they argue that this emotion may be anticipated as a consequence of enacting temptation.^1^

### Resisting Temptation: The Pride of Goal Adherence and the Frustration of Unfulfilled Desires

There are two conflicting emotional responses when resisting temptation: On the one hand, people may feel proud when they act in accordance with their goals and aspirations (MacInnis and Patrick, 2006; Mukhopadhyay and Johar, 2007). On the other hand, people are frustrated by unfulfilled wants (Ortony and Turner, 1990; Anderson and Bushman, 2002; Carver, 2004; Louro et al., 2007). MacInnis and Patrick (2006) described this latter feeling as “deprivation.” However, we do not view deprivation as an emotion *per se* but rather the “lack or denial of something wanted or needed” which *causes* frustration. This definition is consistent with how the term is used in the deprivation literature (e.g., Schachter et al., 1968; Phillips et al., 1984; Grimm et al., 2001; Giordano et al., 2002; Field et al., 2006).^2^ Thus, we suggest that frustration is the self-control emotion that best exemplifies the feeling caused by desires being unfulfilled.

### Scenario-Valence-Motivation (SVM) Taxonomy

**FIGURE 2:**
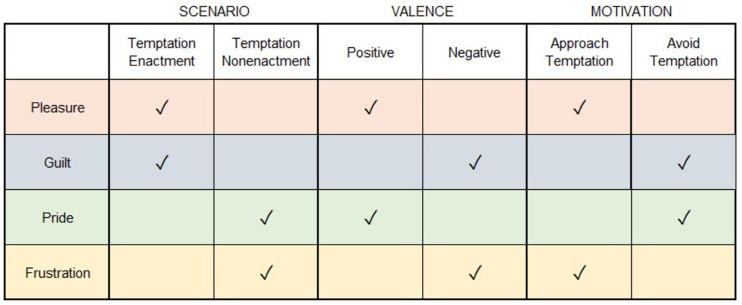
The Scenario-Valence-Motivation (SVM) taxonomy. Each self-control emotion is associated with a unique set of properties across these three dimensions.

From MAESC, we can derive that there are categorical similarities and differences between the core self-control emotions. We highlight these similarities and differences in a taxonomy based on three independent dimensions of each emotion: *scenario*, *valence*, and *motivation* (see Figure 2). *Scenario* refers to whether a given emotion is mainly associated as a consequence of temptation enactment or temptation nonenactment. Pleasure and guilt belong to the temptation-enactment category (*temptation enactment emotions*) and pride and frustration belong to the temptation-nonenactment category (*temptation nonenactment emotions*). *Valence* refers to whether an emotion is positively or negatively valenced. Pleasure and pride belong to the positive-valence category (*positive emotions*) and frustration and guilt belong to the negative-valence category (*negative emotions*). *Motivation* refers to whether the anticipated emotion mainly causes a person to approach or avoid temptation. Pleasure and frustration belong to the approach temptation category (*basic hedonic emotions*) and guilt and pride belong to the avoid temptation category (*self-conscious emotions*) (Eyal and Fishbach, 2010; Trope and Liberman, 2010). We believe that this taxonomy provides a useful conceptual guide and basis to derive hypotheses about the anticipated time courses and relative importance of each anticipated emotion in guiding self-control judgments.

#### Hypotheses

We derive four hypotheses from our model. The first concerns the basic direction of the relationship between each anticipated emotion and self-control judgments. The second concerns the dynamics of the anticipated time course of each emotion – how do the anticipated intensities of pleasure, guilt, pride, and frustration change across time? The third concerns how anticipated emotions are weighted into self-control judgments – how do the anticipated core self-control emotions differ in the extent to which they are weighted into self-control judgments? The fourth concerns the influence of two situational factors – prior self-control exertion and the situational salience of basic hedonic vs. self-conscious emotions.

##### Direction

In line with the model assumptions regarding the motivational function of basic hedonic vs. self-conscious self-control emotions (Figure 2), we hypothesized that:

H1a: Anticipating basic hedonic self-control emotions increases judgment favoring less self-control.
H1b: Anticipating self-conscious self-control emotions increases judgment favoring more self-control.

##### Time courses of anticipated emotions

People not only anticipate how strong their emotions will be (*intensity*) but also how long they will last (*duration*) (Wilson and Gilbert, 2003). We predict that the core self-control emotions have different time courses – less complex, basic hedonic emotions such as pleasure and frustration tend to be relatively short-lived whereas more complex, self-conscious emotions such as guilt and pride tend to be relatively long-lived. The notion that pleasure and frustration tend to be relatively short-lived is suggested, for instance, by dual-systems models that can be used to link these kinds of emotions to a faster, more impulsive mode of responding to the world (Metcalfe and Mischel, 1999; Strack and Deutsch, 2004; Kahneman, 2011). As indicated by the label “self-conscious,” guilt and pride, in contrast, are thought to require self-awareness and arise after self-assessment (Taylor, 1985; Tangney, 1999; Tracy and Robins, 2004) which seems consistent with a slower, more thoughtful and (self-)reflective way of responding. A second, related way of thinking about primary and self-conscious emotions is in terms of concrete vs. abstract: Pleasure and frustration are more “concrete” in that they are involved in monitoring the pursuit of short-term goals, whereas self-conscious emotions are more “abstract” in that they are involved in monitoring the pursuit of long-term goals (Eyal and Fishbach, 2010). In a related vein, basic emotions like pleasure and frustration may be anticipated to be experienced for a shorter time because they are associated with short-term goals (i.e., immediate desires) whereas semotions may be anticipated to be experienced longer because they are associated with long-term goals (Katzir et al., 2010; Shimoni et al., 2016). Consistent with this perspective, Giner-Sorolla (2001) asked people to list their thoughts about idiosyncratic self-control scenarios. Analyzing the written thoughts revealed that people associated negative self-conscious emotions such as guilt more with the longer-term consequences rather than the shorter-term consequences of actions taken in these imagined self-control scenarios. Based on these models and prior data, we hypothesized that these emotions tend to be anticipated as arousing, immediate, and relatively short-lived.

Taken together, we expected that:

H2: Basic hedonic self-control emotions are anticipated to decay relatively quickly compared to self-conscious self-control emotions.

##### Weighting of anticipated emotions

We also hypothesized that the core self-control emotions are differentially weighted in the judgment process. Specifically, we predicted that people would weigh anticipated guilt most when forming self-control judgments for two reasons: First, there is various evidence that people are inclined to simulate temptation enactment (“what if I did?”) more than temptation nonenactment (“what if I didn’t?”) (we refer to this as *enactment orientation*). Desires are action-oriented (Metcalfe and Mischel, 1999; Hofmann and Van Dillen, 2012; Hofmann and Kotabe, 2013) and, furthermore, many social and cognitive neuroscience studies support that mental simulation relies on a neural network designed for representing actions rather than inactions (Grezes and Decety, 2001). Second, people seem to have a robust bias toward weighting negative outcomes in judgment (Rozin and Royzman, 2001), for example, judging losses to loom larger than gains (Tversky and Kahneman, 1991), and “bad” to be stronger than “good” (Baumeister et al., 2001). As a result, people may weight anticipated negative emotions more when forming self-control judgments (we refer to this as *negativity bias*). Because guilt is the only proposed core self-control emotion that is both enactment-oriented and negatively valenced, we hypothesized that people would exhibit a *guilt bias*:

H3a: Anticipated guilt is weighted relatively strongly into self-control judgments (*guilt bias*).

In contrast, because anticipated pride is neither enactment-oriented nor negatively valenced, we hypothesized that people would exhibit *pride neglect*, revealed in anticipated pride being weighed less than the other anticipated emotions into self-control judgments:

H3b: Anticipated pride is weighted relatively weakly into self-control judgments (*pride neglect*).

##### Situational moderators

Perhaps the most important question for practical considerations is *when* anticipated emotions lead to judgments favoring more or less self-control. According to our model, situations decreasing (increasing) weighting of anticipated basic hedonic emotions while increasing (decreasing) weighting of anticipated self-conscious emotions should increase judgments favoring more (less) self-control. As a natural first step in this direction, we manipulated the salience of basic hedonic vs. self-conscious emotions. Specifically, we tested whether self-control judgments would be affected by whether people anticipated all four core self-control emotions vs. only the basic hedonic emotions vs. only the self-conscious emotions. We hypothesized that making the anticipated basic hedonic emotions salient results in judgments favoring less self-control and making the self-conscious emotions salient results in judgments favoring more self-control:

H4a: Increasing the salience of basic hedonic emotions increases judgments favoring less self-control.
H4b: Increasing the salience of self-conscious emotions increases judgments favoring more self-control.

#### The Present Research

We report three studies that tested our hypotheses and validated several aspects of MAESC. In Study 1, we validated that people associate pleasure and guilt with temptation enactment and pride and frustration with temptation nonenactment. In Study 2, we investigated important *characteristics* of the forecasts of each emotion, including their anticipated time courses and their relative weights in guiding self-control judgments. In Study 3, we investigated the effects of increasing the situational salience of basic hedonic vs. self-conscious emotions. In an additional study reported in the Supplementary Material, we also explored the effects of prior self-control exertion as another possible situational moderator variable. The present research was approved by the Internal Review Board of the University of Chicago. Sample size was determined pragmatically and in accordance with available resources by trying to sample at least 80 participants per cell. All data is publicly available on the Open Science Framework at https://osf.io/2bqes/.

## Study 1: Validating Pleasure, Guilt, Pride, and Frustration as Self-Control Emotions

Although considerable evidence already exists to link these emotions to self-control scenarios, we wanted to confirm whether these are appropriate exemplary emotions to focus on in the following studies when assessing the anticipation of these emotions in temptation enactment and nonenactment scenarios. To do this, we presented people with a temptation enactment vs. temptation nonenactment scenario and then had them rate the positive and negative emotions they anticipated to feel strongest in these two scenarios. Participants could select one of the core self-control emotions or had the option to enter an alternative response or no response.

### Materials and Methods

#### Participants and Design

A total of 111 US-based adults recruited on the online labor market Amazon Mechanical Turk (AMT) participated in this two-condition (scenario: temptation enactment vs. temptation nonenactment) within-subjects experiment in return for $1. Participant demographics were not collected in this study.

#### Procedure

Participants first read the following prompt to place their minds in a self-control scenario:

Everyone now and then has to deal with a self-control dilemma. Do I act out temptation and fail to pursue my goal (e.g., eat a cheeseburger and fail to act consistently with my dieting goal)? Or do I resist temptation to pursue a goal instead (e.g., resist the cheeseburger, act according to my dieting goal)? Whether acting out temptation or resisting temptation to pursue a goal instead, one may experience a number of emotions from their decision. We want to hear about your thoughts regarding the emotional consequences of self-control decisions. To that end, we will now ask you to judge which emotions you would anticipate experiencing the strongest after acting out or avoiding temptation. Because we strive for accuracy, it would be best if you answer as honestly as you can. If you believe you would feel emotions not presented in our answer choices, then we have provided the option for you to write in an alternative response.

Participants were then instructed to select one positive emotion and one negative emotion they anticipated they would experience strongest in a temptation enactment scenario and a temptation nonenactment scenario. These instructions read (manipulated text in italics):

“Below, we list a number of emotions that may be experienced *after acting out temptation and failing to pursue a self-control goal*/*after avoiding temptation to pursue a self-control goal instead*. Which positive and negative emotions do you anticipate you would experience strongest?”

The order of the scenarios (temptation enactment vs. temptation nonenactment) was randomized. The choice options included the four core self-control emotions – pleasure, guilt, pride and frustration – in randomized order, and four additional text boxes to enter alternative responses. In addition, they were allowed to not vote for any of the options. We expected that participants would select one of the core self-control emotions (e.g., pleasure) if they had the general notion that they would experience something *like* that emotion (e.g., fun, joy, satisfaction, happiness). Importantly, this behavior would not be problematic as our intention was to identify *exemplary* emotions for the study of anticipated emotions in self-control, rather than to distinguish between all of the specific positive and negative emotion possibly involved.

### Results and Discussion

**FIGURE 3:**
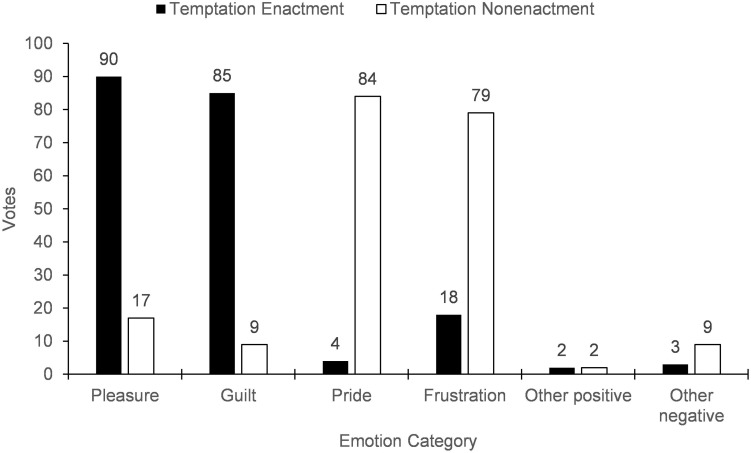
Participant votes on emotions likely to be experienced after enacting or not enacting temptation (Study 1).

A repeated-measures logistic regression confirmed that the number of votes for anticipated pleasure, *B* = 3.17, Wald χ^2^ = 66.40, *p* < 0.001, 95% CI [2.40, 3.93], *OR* = 23.69, and anticipated guilt, *B* = 3.61, Wald χ^2^ = 71.71, *p* < 0.001, 95% CI [2.78, 4.45], *OR* = 36.97, was significantly higher in the temptation enactment scenario whereas the number of votes for anticipated pride, *B* = −4.42, Wald χ^2^ = 54.34, *p* < 0.001, 95% CI [−5.60, −3.25], *OR* = 83.10, and anticipated frustration, *B* = −2.55, Wald χ^2^ = 51.21, *p* < 0.001, 95% CI [−3.24, −1.85], *OR* = 12.81, was significantly higher in the temptation nonenactment scenario (see Figure 3). The number of votes for “other negative emotions” also significantly differed by scenario, *B* = −1.16, Wald χ^2^ = 5.68, *p* = 0.017, 95% CI [−2.11, −0.21], *OR* = 3.19, but the number of votes for “other positive emotions” did not – overall, however, there were far fewer votes for the “other” categories (16 total vs. 386 total for the self-control emotions), so we do not interpret these results further. The frequencies per self-control emotion supported our decision to associate anticipated pleasure and guilt with the temptation enactment scenarios and anticipated pride and frustration with the temptation nonenactment scenarios in the following studies.

## Study 2: Basic Characteristics

The purpose of Study 2 was to provide a comprehensive test of our first three hypotheses. To do this, we asked people in this study to estimate the anticipated intensity of each core self-control emotion across time in a variety of prototypical self-control scenarios before they reported self-control judgments. The fine-grained nature of the data obtained allowed us to investigate the anticipated time courses of basic hedonic vs. self-conscious emotion (H2). We also tested our hypotheses regarding directional effects (H1a/b) and weighting of anticipated emotions for self-control judgments (H3a/b). Specifically, we sought to (a) test the model assumption that the anticipated core self-control emotions can independently guide self-control judgments in the expected direction and (b) test whether anticipated guilt would be weighted most and anticipated pride would be weighted least into self-control judgments, as predicted by enactment orientation and negativity bias.

### Materials and Methods

#### Participants and Design

A total of 128 US-based adults (54 men, 74 women) recruited on AMT participated in this cross-sectional study in return for $3. Ages were recorded categorically: 13 participants were between the ages of 18 and 21, 18 were between the ages 22 and 25, 26 were between the ages of 26 and 30, 28 were between the ages of 30 and 40, 26 were between the ages of 41 and 50, 13 were between the ages of 51 and 60, and 3 were over the age of 61. 111 participants identified as White/Caucasian, 8 identified as Asian/Asian American, 7 identified as Black/African American, 6 identified as Hispanic/Latino, 4 identified as Native American/Alaska Native, and 1 identified as “other”.

#### Materials

To trace the time courses of participants’ *anticipated* emotions, we developed a task that measured not only people’s predictions of their immediate emotional reactions, but also the longer-term temporal dynamics of each anticipated emotion. We call this task the Anticipated Response to Affective Events Task (AReA Events Task). It is a flexible tool for assessing the intensity and duration dimensions of any anticipated emotion at any level of granularity.

The task was designed in JavaScript for online use. Although we used it specifically to study anticipated emotions in self-control scenarios, it can be modified to address a variety of questions about the anticipation of emotions that take into account intensity and duration. In the version of the task used in this study, participants were randomly presented four vignettes portraying prototypical self-control scenarios. To sample a broad variety of self-control scenarios, we used food, alcohol, sex, and aggression temptation scenarios (for methodological details on the vignettes see Supplementary Material). At the end of each vignette, participants were reminded of the self-control dilemma – they could act on temptation or they could try to resist temptation. After each vignette, participants were asked to anticipate the intensity and duration of the emotions associated with the decision to enact or not enact temptation using interactive slider scales, with time “0” indicating the moment a decision was made (see Supplementary Figure 1). Pleasure and guilt were paired together under a temptation enactment header (“If I [enacted temptation]…”) and pride and frustration were paired together under a temptation nonenactment header (“If I [avoided temptation]…”). Pair order and order within each pair were randomized. Each emotion was accompanied by vertical slider scales, lined up side-by-side, so as to create a y-axis that captured the anticipated intensity dimension and an x-axis that captured the anticipated duration dimension. For anticipated intensity, values ranged from 0 (no intensity) to 100 (full intensity). For anticipated duration, values ranged from 0 to 60 min after temptation enactment or temptation nonenactment, with a slider scale at each 10 min interval. Interval length and maximum duration were based on pretesting the materials on members of our lab, due to lack of previous research to guide these decisions.

#### Procedure

Participants first completed the AReA Events Task as described above, then they provided ratings reflecting their self-control judgments. Rather than directly asking participants how likely they would be to act in a self-controlled manner in a given scenario, we asked them how likely they would be to enact temptation because past research suggests that self-reports are more inaccurate for positive self-evaluations due to self-enhancement and/or self-presentation (Paulhus, 1998). As self-control is virtuous (Baumeister and Juola Exline, 1999; Hofmann et al., 2014), we wanted to avoid making it too salient in the rating task. These ratings were made using a seven-point likelihood scale ranging from *very unlikely* to *very likely* and were reverse-scored to assess self-control judgments.

### Data Analytic Procedures

#### Aggregating Anticipated Emotions Data

To integrate the intensity and duration dimension of anticipated emotions, we summed the values of the anticipated emotional intensities across time to estimate the area under the curve of each anticipated emotion trajectory (see Supplementary Table 1 for descriptive statistics for each anticipated emotion area across self-control scenarios), We refer to this anticipated emotion area measure in shorthand as “anticipated [core self-control emotion]” (e.g., “anticipated pleasure predicts X” would be shorthand for “the anticipated pleasure area value predicts X”).

#### Multilevel Analyses

Self-control scenarios were nested within participants. To aggregate data across these scenarios, weighting analysis was conducted using a linear mixed-effects model, with scenarios effect-coded and a random intercept for each participant. We statistically controlled for scenarios and all two-way interactions between scenarios and the anticipated emotions in order to provide a general picture of the role of anticipated emotions in self-control scenarios. Trajectory analysis was also conducted using multilevel modeling, with time predicting anticipated emotion intensities averaged across scenarios and a random intercept for each participant. One unit of time was equal to an interval of 10 min. For all simple slopes analysis, *H*_0_: *B* = 0 and *H*_1_: *B* ≠ 0. All continuous independent and dependent variables were left in their original units in the trajectory analysis for interpretability and were standardized in weighting analysis to allow for comparisons.

### Results and Discussion

#### Trajectories

**FIGURE 4:**
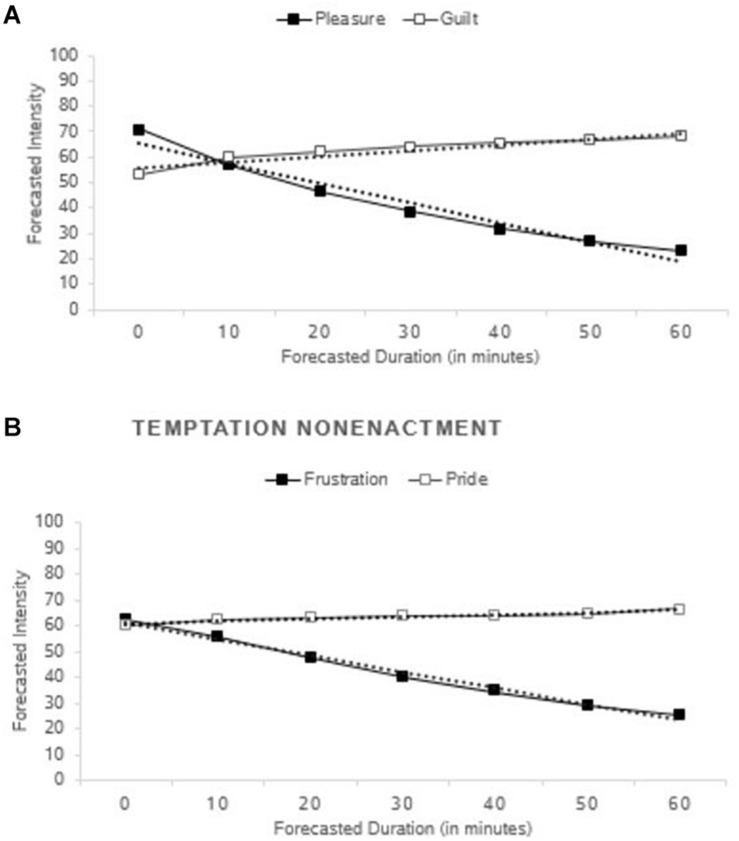
Trajectories of anticipated enactment **(A)** and nonenactment **(B)** emotions in Study 2. Dotted lines represent linear estimates based on multilevel modeling and solid lines represent raw means.

Supporting H2, pleasure and frustration were anticipated to decay faster (pleasure, *B* = −7.77; *p* < 0.001, 95% CI [−8.21, −7.34]; frustration, *B* = −6.31; *p* < 0.001, 95% CI [−6.69, −5.92]) (see Figure 4A) than pride and guilt, which were anticipated to intensify over time (pride, *B* = 0.90, *p* < 0.001, 95% CI [0.54, 1.26]; guilt, *B* = 2.24; *p* < 0.001, 95% CI [1.85, 2.63]) (see Figure 4B). Moderated regression using a dummy-coded variable for self-conscious emotions confirmed that the difference in slopes between anticipated basic hedonic emotions and anticipated self-conscious emotions was significant, *B* = −8.61, *p* < 0.001, 95% CI [8.02, 9.19]. These results suggest that people anticipate pleasure to decay faster than guilt after temptation enactment and frustration to decay faster than pride after temptation nonenactment.

#### Weighting

We regressed self-control judgments on the anticipated emotion variables (see Table 1 for a summary of the judgment weights of each anticipated self-control emotion across all studies). As predicted, the core anticipated emotions independently predicted self-control judgments. Anticipated pleasure, β = −0.24, *p* < 0.001, 95% CI [−0.32, −0.16], and anticipated frustration, β = −0.18, *p* < 0.001, 95% CI [−0.26, −0.10], predicted judgments favoring less self-control whereas anticipated guilt, β = 0.39, *p* < 0.001, 95% CI [0.31, 0.47], and anticipated pride, β = 0.11, *p* = 0.004, 95% CI [0.35, 0.19], predicted judgments favoring more self-control, supporting H1a and H1b, respectively. Comparing these results to the trajectory results, it seems that people anticipate similar amounts of pride as guilt but take the latter more into consideration when forming self-control judgments.

**TABLE 1:** Weighting of anticipated emotions into self-control judgments across all studies.

		**Pleasure**		**Guilt**		**Pride**		**Frustration**	
					
		**β**	***SE***	**β**	***SE***	**β**	***SE***	**β**	***SE***
**Study 2** (characteristics)	–	−0.24	0.04	0.39	0.04	0.11	0.04	−0.18	0.04
**Study 3** (salience)	PR	−0.27	0.11	–	–	–	–	−0.20	0.11
	SC	–	–	0.70	0.21	0.14	0.20	–	–
	ALL	−0.18	0.09	0.38	0.09	0.03	0.10	−0.14	0.10
Supplementary Study 4 (prior self-control exertion)	Control	−0.12	0.06	0.52	0.07	0.03	0.07	−0.16	0.07
	Prior self-control exertion	−0.34	0.07	0.17	0.07	0.20	0.07	−0.19	0.07
**Overall**		−0.23		0.43		0.10		−0.17	

**Positive regression coefficients indicate judgment favoring temptation nonenactment, negative coefficients indicate judgments favoring temptation enactment. PR, only the basic hedonic self-control emotions made situationally salient; SC, only the self-conscious self-control emotions made situationally salient; ALL, all core self-control emotions made situationally salient. The NONE condition is now shown because there were no coefficients to report.**

Consistent with H3a, anticipated guilt most strongly predicted self-control judgments. Contrasts comparing absolute beta coefficients indicated that the anticipated guilt effect was significantly larger than the average effect of the other three anticipated emotions, β_dif_ = 0.21, *p* < 0.001, 95% CI [0.10, 0.31], and also significantly larger than the individual effects of anticipated pleasure, β_dif_ = 0.15, *p* = 0.023, 95% CI [0.02, 0.27], anticipated pride, β_dif_ = 0.27, *p* < 0.001, 95% CI [0.14, 0.41], and anticipated frustration, β_dif_ = 0.21, *p* = 0.001, 95% CI [0.10, 0.32].

Consistent with H3b, anticipated pride was the weakest predictor of self-control judgments. Contrasts comparing absolute beta coefficients indicated that the anticipated pride effect was significantly smaller than the average effect of the other three anticipated emotions, β_dif_ = −0.16, *p* = 0.001, 95% CI [−0.25, −0.07], and also significantly smaller than the individual effects of anticipated pleasure, β_dif_ = −0.13, *p* = 0.013, 95% CI [−0.23, −0.03], and anticipated guilt (see above), but not anticipated frustration, β_dif_ = −0.07, *p* = 0.20, 95% CI [−0.18, 0.04].

We theorized that relatively strong weighting of anticipated guilt and relatively weak weighting of anticipated pride may be due to *enactment orientation* and *negativity bias*. In support of enactment orientation, a linear contrast indicated that the sum of the absolute coefficients of the enactment emotions was significantly greater than the sum of the absolute coefficients of the nonenactment emotions, β_dif_ = 0.33, *p* < 0.001, 95% CI [0.16, 0.51]. In support of negativity bias, a linear contrast indicated that the sum of the absolute coefficients of the negative emotions was significantly greater than the sum of the absolute coefficients of the positive emotions, β_dif_ = 0.21, *p* = 0.034, 95% CI [0.02, 0.41].

Study 2 provides several insights into the role of anticipated emotions in the formation of self-control judgments. First, using a novel task – the AReA Events Task – to track anticipated emotional intensity over time, we showed that people anticipate that basic hedonic emotions would decay faster than self-conscious emotions in self-control scenarios. Second, we showed that all four anticipated emotions can independently predict self-control judgments – anticipated basic hedonic emotions independently predicted judgments favoring less self-control whereas anticipated guilt and pride independently predicted judgments favoring more self-control. These findings corroborate a core assumption of MAESC – that the anticipation of each of these emotions can play a significant role in guiding self-control judgments. Further, our analysis revealed that the anticipated core self-control emotions were differentially weighted into self-control judgments – the weighting pattern is characterized by relatively strong weighting of anticipated guilt and relatively weak weighting of anticipated pride. The results also provide some support that enactment orientation and negativity bias account for these tendencies.

## Study 3: Effects of the Situational Salience of Basic Hedonic Vs. Self-Conscious Emotions

The main purpose of Study 3 was to investigate another possible situational influence: the situational salience of basic hedonic vs. self-conscious emotions. Specifically, we tested how the situational salience of basic hedonic vs. self-conscious emotions affects the weighting of those anticipated emotions into self-control judgments. We also included a condition in which all four core self-control emotions were situationally salient as this matches the conditions of Study 2, and a condition in which no emotions were made situationally salient as a control condition. Due to the increased focus of attention brought about by high salience, we predicted that making self-conscious emotions (basic hedonic emotions) situationally salient would increase people’s weighting of those emotions into self-control judgments, resulting in judgments favoring more (less) self-control. We also predicted that increasing the situational salience of all four emotions would result in an intermediate outcome. Another purpose of this study was to extend our study to a new population – a community sample from Chicago.

### Materials and Methods

#### Participants and Design

A total of 242 adults (163 men, 79 women) from the downtown Chicago, IL community were recruited and participated in this four-condition (situational salience: no emotions vs. only basic hedonic emotions vs. only self-conscious emotions vs. all core self-control emotions) between-subjects experiment in return for $6. Ages ranged from 18 to 70 (*M* = 33.68, *SD* = 12.54). 131 participants identified primarily as Black/African American, 67 identified as White/Caucasian, 16 identified as Hispanic/Latino, 10 identified as Asian/Asian American, 4 identified as Native American/Alaska Native, and 13 identified as other. Thus, this sample was substantially different in terms of ethnicity than the samples observed in the previous studies.

#### Procedure

Participants were randomly assigned either to the “no salient emotions” (NONE), “salience of basic hedonic emotions” (PR), “salience of self-conscious emotions” (SC), or “salience of all core self-control emotions” (ALL) condition. All participants completed versions of the AReA Events Task that varied on which emotions participants were instructed to anticipate. The vignettes were identical across conditions. As in Study 2, each participant was randomly presented two of the four vignettes. In the NONE condition, participants read each vignettes before reporting their self-control judgment without doing the AReA Events Task. This condition best captured self-control judgments following the spontaneous anticipation of emotions, since there were no explicit requests to anticipate emotions. In the PR condition, participants read the temptation vignettes then did a version of the AReA Events Task in which they anticipated pleasure and frustration (presentation order randomized) but not guilt and pride. In the SC condition, participants read the temptation vignettes then did a version of the AReA Events Task in which they anticipated guilt and pride (presentation order randomized) but not pleasure and frustration. In the ALL condition, participants read the temptation vignettes then did the version of the AReA Events Task in which they anticipated all four core self-control emotions, as in Study 2. Subsequently, all participants reported their self-control judgments.

### Data Analytic Procedures

The data analytic procedures were the same as those used to analyze data from Study 2 except that we entered a dummy coded condition variables (NONE as reference condition) and their two-way interactions with scenario into the main effects models.

### Results and Discussion

#### Main Effects

**FIGURE 5:**
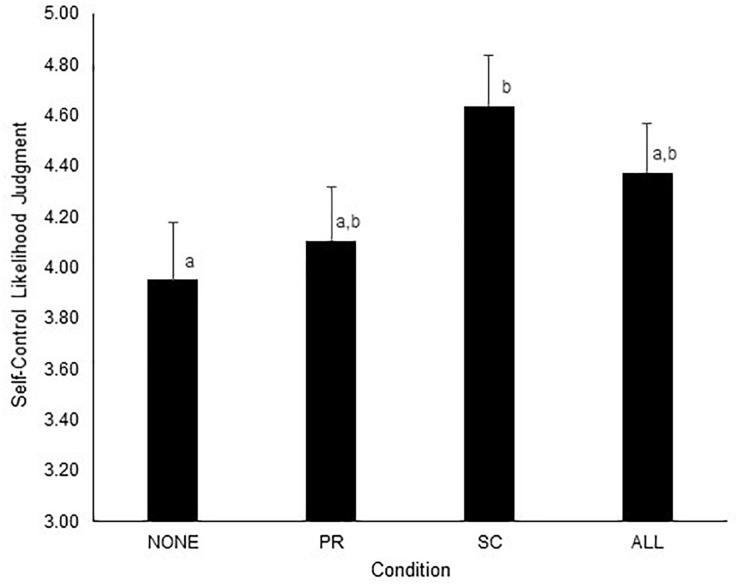
Effects of increasing the situational salience of different anticipated emotions on self-control judgments in Study 3. NONE, no core self-control emotions made situationally salient; PR, only the basic hedonic self-control emotions made situationally salient; SC, only the self-conscious self-control emotions made situationally salient; ALL, all core self-control emotions made situationally salient. Self-control judgments were measured with a 1–7 likelihood scale (very unlikely to very likely). Error bars indicate +1 *SE*. Means not sharing the same subscript are significantly different from each other at *p* < 0.05.

Using the NONE condition as a baseline, increasing the situational salience of the self-conscious emotions resulted in judgments favoring more self-control, *B* = 0.69, *p* = 0.025, 95% CI [0.09, 1.29] (see Figure 5), supporting H4b. However, increasing the situational salience of basic hedonic emotions did not result in judgments favoring less self-control, *B* = 0.15, *p* = 0.623, 95% CI [−0.46, 0.77], contrary to H4a. Moreover, increasing the situational salience of all four core self-control emotions, *B* = 0.42, *p* = 0.161, 95% CI [−0.17, 1.02], did not significantly change self-control judgments as compared to baseline.

Hence, this study shows that making self-conscious emotions situationally salient by having people explicitly forecast only pride and guilt can positively impact self-control decision-making. These findings accord well with prior work on anticipated self-conscious affect (Abraham and Sheeran, 2003; Patrick et al., 2009), and should be considered when designing interventions focused on adjusting their use of self-control. We can only speculate *post hoc* as to why the manipulation of situational salience of the basic hedonic emotions did not have a significant effect in the predicted direction, but it may be due to basic hedonic emotions being more salient at baseline compared to the self-conscious emotions (Giner-Sorolla, 2001). The results of the study by Giner-Sorolla suggest that all four of the core self-control emotions are considered in self-control contexts, but that basic hedonic emotions are relatively more salient, perhaps because of their concreteness (Eyal and Fishbach, 2010).

#### Trajectories

In the ALL condition, we again found that the basic hedonic emotions were anticipated to decay faster than the self-conscious emotions, which were anticipated to intensify: anticipated pleasure, *B* = −4.23, *p* < 0.001, 95% CI [−5.07, −3.39]; anticipated frustration, *B* = −2.77, *p* < 0.001, 95% CI [−3.52, −2.02]; anticipated guilt, *B* = 2.24, *p* < 0.001, 95% CI [1.48, 3.00]; and anticipated pride, *B* = 0.99, *p* = 0.010, 95% CI [0.24, 1.74]. Moderated regression using a dummy-coded variable for self-conscious emotions confirmed that the difference in slopes between anticipated basic hedonic emotions and anticipated self-conscious emotions was again significant, *B* = −4.51, *p* < 0.001, 95% CI [3.74, 5.28]. Similarly, in the PR condition, pleasure, *B* = −2.94, *p* < 0.001, 95% CI [−3.77, −2.11], and frustration, *B* = −1.73, *p* < 0.001, 95% CI [−2.53, −0.92], were anticipated to decay, whereas in the SC condition, guilt, *B* = 1.59, *p* < 0.001, 95% CI [0.86, 2.31], and pride, *B* = 1.38, *p* = 0.001, 95% CI [0.71, 2.05], were anticipated to intensify. All of these results support H2.

#### Weighting

We regressed self-control judgments on the anticipated core self-control emotions. In the ALL condition, anticipated pleasure marginally predicted judgments favoring less self-control and anticipated guilt significantly predicted judgments favoring more self-control, β = -0.18, *p* = 0.058, 95% CI [−0.37, 0.01] and β = 0.38, *p* < 0.001, 95% CI [0.20, 0.57], respectively (see Table 1 for comparisons with the other conditions) (anticipated pride and anticipated frustration were not significant predictors in this case, β = 0.03, *p* = 0.749, 95% CI [−0.17, 0.23] and β = −0.14, *p* = 0.130, 95% CI [−0.33, 0.04], respectively). We again found evidence of the importance of anticipated guilt. Contrasts comparing absolute beta coefficients indicated that the anticipated guilt effect was significantly larger than the average effect of the other three anticipated emotions, β_dif_ = 0.26, *p* = 0.033, 95% CI [0.02, 0.51], and significantly larger than the individual effects of anticipated pride, β_dif_ = 0.35, *p* = 0.041, 95% CI [0.02, 0.69], and anticipated frustration, β_dif_ = 0.24, *p* = 0.040, 95% CI [0.01, 0.47]. It was also descriptively but not significantly larger than the individual effect of anticipated pleasure, β_dif_ = 0.20, *p* = 0.177, 95% CI [−0.09, 0.50]. As for the relatively weak importance of anticipated pride, contrasts comparing absolute beta coefficients indicated that the anticipated pride effect was marginally smaller than the average effect of the other anticipated emotions, β_dif_ = −0.20, *p* = 0.077, 95% CI [−0.43, 0.02], and also significantly smaller than the individual effects of anticipated guilt (see above), but not significantly smaller than the individual effects of anticipated pleasure, β_dif_ = −0.15, *p* = 0.174, 95% CI [−0.36, 0.07], and anticipated frustration, β_dif_ = −0.11, *p* = 0.422, 95% CI [−0.39, 0.16]. The significant results here also support H1a, H1b, H3a, and H3b. In the PR condition, anticipated pleasure and anticipated frustration marginally to significantly predicted judgments favoring less self-control: β = −0.27, *p* = 0.015, 95% CI [−0.49, −0.05] and β = −0.20, *p* = 0.058, 95% CI [−0.41, 0.01], respectively. In the SC condition, anticipated guilt predicted judgments favoring more self-control, β = 0.70, *p* = 0.001, 95% CI [0.28, 1.11], but anticipated pride did not, β = 0.14, *p* = 0.488, 95% CI [−0.25, 0.53], consistent with the previous studies which demonstrated relatively strong weighting of anticipated guilt and relatively weak weighting of anticipated pride. The results in this study were not as strong as in Study 2. However, as can be seen in Table 1, the weighting of the self-control emotions was remarkably consistent in this study compared with the previous studies despite observing a substantially different participant sample.

## General Discussion

People experience desires about half the time they are awake and about half the time these desires are in conflict with some higher-order goal (see Hofmann et al., 2012). These desires come in diverse shapes and forms, from desires for food and drinks to media consumption and socialization to sex and shopping. The judgment process involved in deciding whether to enact desires in conflict with long-term goals is one that has puzzled great thinkers throughout history. The present theoretical and empirical analysis suggests that an important but underappreciated part of this judgment process is the anticipation of mixed and conflicting emotions. Our study sheds light on several aspects of this process, thus providing both theoretical and practical insights.

In Study 1, we corroborated that pleasure and guilt exemplify the main emotional experiences associated with temptation enactment and pride and frustration exemplify the main emotional experiences associated with temptation nonenactment. The theoretical rationale is that temptation enactment causes both desire fulfillment (associated with pleasure) and goal violation (associated with guilt), whereas temptation nonenactment causes both desire nonfulfillment (associated with frustration) and goal adherence (associated with pride). This basic framework is useful for understanding how desires and goals elicit (anticipated) emotional responses.

Results from Study 2 support the main propositions of MAESC. First, people anticipate that basic hedonic self-control emotions – pleasure and frustration – will decay faster than self-conscious self-control emotions – guilt and pride. Second, people differentially weigh each of these emotions into self-control judgments. Specifically, people display a guilt bias and pride neglect as reflected in their weighting patterns favoring guilt the most and pride the least, which was also replicated in Study 3 and Supplementary Study 4. Anticipating pride from goal adherence tunes judgment toward favoring more self-control. In situations where self-controlled behavior is desirable, those in charge of designing choice environments could benefit from this knowledge by creating environments that emphasize the importance of the prideful experience of sticking to one’s goals (Thaler and Sunstein, 2008). In situations where impulsive behaviors are desirable, it may be useful to deemphasize the importance of the guilt-ridden experience of violating one’s goals, though the ethical implications should be carefully considered.

Study 3 provides direct empirical evidence that self-control judgments can be manipulated by interventions targeting the salience of specific self-control emotions. Compared to when no emotions are made salient, increasing the situational salience of self-conscious emotions tuned judgments more in favor of self-control. This is of practical importance because it demonstrates that interventions directly targeting anticipated emotions can significantly affect their self-control judgments. When an abstinent alcoholic is faced with a menu offering whiskey or soda, a choice environment increasing the salience of self-conscious emotions could turn his decision toward a soda sans whiskey rather than a whiskey soda, a decision that could prevent a series of regretful decisions.

### Future Directions for Research

Although MAESC describes anticipated emotions in self-control scenarios in particular, the general framework of the model could be extended to other mixed-emotions dilemmas. In the general framework, multiple prospects are simulated, resulting in the anticipation of emotions associated with those prospects, which are in turn weighted into judgments about how one would behave in a given scenario. This general framework could be applied to situations in which people assess morality or risk. For example, imagine the sort of “trolley problem” consumers and lawmakers face at the dawn of the age of the autonomous vehicle: In a life or death situation, does an autonomous vehicle risk the life of the pedestrian or the driver? It depends on how developers program the vehicle’s automation algorithm. Casting support for a driver-friendly vs. pedestrian-friendly automation algorithm may elicit the anticipation of positive affect associated with the scenario of saving a life while also eliciting the anticipation of negative affect associated with the scenario of ending a life. The structure of the judgment problem here is similar to the judgment problem in self-control dilemmas. There are alternative scenarios and each is associated with positive and negative affect. Identifying specific anticipated emotions involved, how they are weighted in moral judgment, and the factors that moderate these weights would be useful for understanding how people decide which scenario is right or wrong.

This work also introduces a novel task to study anticipated emotions in a variety of additional decision-making contexts – the AReA Events Task. With this task, one can capture and integrate both the intensity and duration dimensions of anticipated emotions, affording the opportunity to trace the temporal dynamics of individual anticipated emotions. From there, one may derive various measures concerning anticipated emotions such as measures of anticipated emotional intensity across time and intensification and decay rates. Furthermore, the AReA Events Task is flexible as it may be adapted to assess the anticipation of single or multiple emotions and can take any intensity and duration values. Such flexibility provides the task with the potential to answer many unanswered questions about the role of anticipated emotions in guiding human judgment.

In the present work, we tried to better understand how key anticipated emotions guide self-control judgments. There are limitations and open questions that can guide future research in this direction: Regarding limitations, the present work is clearly limited by the hypothetical nature of the scenarios used. While providing a relatively high degree of control, it would seem important for future research to replicate our findings in the context of more ecologically valid settings that induce anticipated emotions when confronted with typical self-control scenarios involving real tempting stimuli in the lab. In terms of open questions, are there individual differences (e.g., cultural background, learning history) that moderate these effects? What is the role of anticipated emotions in special case scenarios such as those involving drug or gambling addiction? Regarding the design of environments, how can marketers design low-cost interventions or “nudges” (Thaler and Sunstein, 2008) targeting anticipated emotions to significantly impact human decision making? One domain which has garnered special attention in consumer research is impulsive buying (Rook, 1987) in which self-control research is of particular importance (Hoch and Loewenstein, 1991). Consumers are sometimes hit with a powerful, compulsive, and spontaneous urge to buy, leaving them feeling helpless under the power of desire. Sometimes it would be in the better interest of impulse buyers to *change their mind* (Rook, 1987). The results of Study 3 suggest that interventions targeting avoid-oriented or self-conscious anticipated emotions could be effective. Orienting consumers toward anticipating self-conscious emotions may increase their weighting into their self-control judgments, resulting in increased motivation to self-control. Savings is another domain in which orienting consumers toward anticipating self-conscious emotions could be beneficial (Thaler and Shefrin, 1981; Shefrin and Thaler, 1988). According to Shefrin and Thaler, consumer self-control underlies the national savings rate, and savings has been described as the “mirror image of consumption” (Hoch and Loewenstein, 1991). In terms of saving interventions, it may also be useful to target anticipated self-conscious emotions.

In particular, we believe that the neglect of anticipated pride deserves special attention. It is even a curious parallel that in the social and personality psychology literature, there is little attention paid to pride as compared with guilt (see Tracy and Robins, 2007). Why do people discount this anticipated emotion when forming self-control judgments? Research on pride distinguishes between authentic and hubristic pride, with the former being attributed to specific actions or accomplishments and the latter being coming from a global evaluation of the self (Tracy and Robins, 2007). People may be averse to placing importance on hubristic pride, insofar as hubris is a socially undesirable trait. For example, people may especially be averse to placing importance on hubristic pride after spending time in cultures in which such pride is considered a sin. In the U.S., where we conducted our study, and where ∼75% of American adults identify as Christians (“Percentage of Christians in U.S. Drifting Down, but Still High,” 2015), hubristic pride may be associated with sin. It is possible that participants in our study attributed the scenario of temptation nonenactment with hubristic pride, and thus discounted it when forming self-control judgments. We did not direct our participants to think in this way, so it opens up an interesting avenue for future research, which would be to test whether directing or manipulating people to think of pride from temptation nonenactment in an authentic vs. hubristic way to see whether it shifts weighting of pride into self-control judgments.

## Conclusion

People take into account the feelings they anticipate when they make choices. When asking themselves everyday questions such as what they should eat or drink, where they should shop, and what should they buy, they anticipate how they will feel in possible futures associated with each choice. When desires and goals are at odds, the feelings they anticipate are mixed and conflicting, which presents a dilemma concerning how strongly they will experience each emotion anticipated, how long it will last, and how important that is for their decision. Here, we developed a theoretical model of the role of anticipated emotions in such self-control scenarios, and empirically tested its main propositions. Our theoretical and empirical analysis yields useful insights for understanding how people use anticipated feelings to guide their choices.

## Ethics Statement

This study was carried out in accordance with the recommendations of the APA Ethical Principles of Psychologists and Code of Conduct with written informed consent from all subjects. All subjects gave written informed consent in accordance with the Declaration of Helsinki. The protocol was approved by the Internal Review Board of the University of Chicago.

## Author Contributions

HK and WH contributed conception and design of the study, performed the statistical analysis, and wrote the manuscript. FR provided critical revision inputs. All authors contributed to manuscript revision, and read and approved the submitted version.

## Conflict of Interest Statement

The authors declare that the research was conducted in the absence of any commercial or financial relationships that could be construed as a potential conflict of interest.

## Footnotes

1 The question arises whether one should focus on guilt or shame. In lay use, these terms are often used interchangeably. As evidence, Hynie et al. (2006) found that self-reports of anticipated guilt and shame correlated closely (α = 0.94). Guilt arises from negative evaluations of specific behaviors or transgressions whereas shame arises specifically from public disapproval and, more broadly, a global negative evaluation of the self (Lewis, 1971). Because our theoretical model focuses on the anticipation of specific behavioral transgressions (i.e., the enactment of temptations), we focus our discussion on anticipated guilt.

2 Another important emotion to consider here is regret (see MacInnis and Patrick, 2006). However, regret is associated with both inactions and actions (Summerville, 2011). That is, people may feel regret because of unfulfilled desires or they may feel regret because of violating their goals. As evidence, in an analysis of a large experience sampling study on everyday temptations, it was found that self-reported regret was relatively high whether people enacted or did not enact temptation (Hofmann et al., 2013). Because the source of anticipated regret may be relatively ambiguous, we decided to focus on anticipated frustration, which is specifically associated with unfulfilled wants.

## References

[B1] Abraham C. Sheeran P. (2003). Acting on intentions: the role of anticipated regret. *Br. J. Soc. Psychol.* 42 495–511. 10.1348/014466603322595248 14715114

[B2] Ajzen I. (1991). The theory of planned behavior. *Organ. Behav. Hum. Decis. Process.* 50 179–211. 10.1016/0749-5978(91)90020-T

[B3] Anderson C. A. Bushman B. J. (2002). Human aggression. *Annu. Rev. Psychol.* 53 27–51. 11752478 10.1146/annurev.psych.53.100901.135231

[B4] Bagozzi R. P. Dholakia U. M. Basuroy S. (2003). How effortful decisions get enacted: the motivating role of decision processes, desires, and anticipated emotions. *J. Behav. Decis. Making* 16 273–295. 10.1002/bdm.446

[B5] Bagozzi R. P. Pieters R. (1998). Goal-directed emotions. *Cogn. Emot.* 12 1–26. 10.1080/026999398379754

[B6] Baumeister R. F. Bratslavsky E. Finkenauer C. Vohs K. D. (2001). Bad is stronger than good. *Rev. Gen. Psychol.* 5 323–370. 10.1037//1089-2680.5.4.323

[B7] Baumeister R. F. Heatherton T. F. Tice D. (1994). *Losing Control: How and Why People Fail at Self-Regulation.* San Diego, CA: Academic Press.

[B8] Baumeister R. F. Juola Exline J. (1999). Virtue, personality, and social relations: self-control as the moral muscle. *J. Pers.* 67 1165–1194. 10.1111/1467-6494.00086 10637991

[B9] Berrios R. Totterdell P. Kellett S. (2015). Investigating goal conflict as a source of mixed emotions. *Cogn. Emot.* 29 755–763. 10.1080/02699931.2014.939948 25040183

[B10] Carver C. S. (2004). “Self-regulation of action and affect,” in *Handbook of Self-Regulation: Research, Theory, and Applications*, eds Baumeister R. F. Vohs K. D. (New York, NY: Guilford Press), 13–39.

[B11] Eyal T. Fishbach A. (2010). Do global and local systems feel different? *Psychol. Inq.* 21 213–215. 10.1080/1047840x.2010.503184

[B12] Field M. Santarcangelo M. Sumnall H. Goudie A. Cole J. (2006). Delay discounting and the behavioural economics of cigarette purchases in smokers: the effects of nicotine deprivation. *Psychopharmacology* 186 255–263. 10.1007/s00213-006-0385-4 16609902

[B13] Freud S. (1920). *A General Introduction to Psychoanalysis.* New York, NY: Horace Liveright.

[B14] Giner-Sorolla R. (2001). Guilty pleasures and grim necessities: affective attitudes in dilemmas of self-control. *J. Pers. Soc. Psychol.* 80 206–221. 10.1037//0022-3514.80.2.206 11220441

[B15] Giordano L. A. Bickel W. K. Loewenstein G. Jacobs E. A. Marsch L. Badger G. J. (2002). Mild opioid deprivation increases the degree that opioid-dependent outpatients discount delayed heroin and money. *Psychopharmacology* 163 174–182. 10.1007/s00213-002-1159-2 12202964

[B16] Grezes J. Decety J. (2001). Functional anatomy of execution, mental simulation, observation, and verb generation of actions: a meta-analysis. *Hum. Brain Mapp.* 12 1–19. 10.1002/1097-0193(200101)12:1<1::aid-hbm10>3.0.co;2-v 11198101 PMC6872039

[B17] Grimm J. W. Hope B. T. Wise R. A. Shaham Y. (2001). Neuroadaptation: incubation of cocaine craving after withdrawal. *Nature* 412 141–142. 10.1038/35084134 11449260 PMC2889613

[B18] Hoch S. J. Loewenstein G. F. (1991). Time-inconsistent preferences and consumer self-control. *J. Consum. Res.* 17 492–507. 10.1086/208573

[B19] Hofmann W. Baumeister R. F. Förster G. Vohs K. D. (2012). Everyday temptations: an experience sampling study of desire, conflict, and self-control. *J. Pers. Soc. Psychol.* 102 1318–1335. 10.1037/a0026545 22149456

[B20] Hofmann W. Kotabe H. P. (2013). “Desire and desire regulation: Basic processes and individual differences,” in *Handbook of Emotion Regulation*, 2nd Edn, ed. Gross J. J. (New York, NY: Guilford Press).

[B21] Hofmann W. Kotabe H. P. Luhmann M. (2013). The spoiled pleasure of giving in to temptation. *Motiv. Emot.* 37 733–742. 10.1007/s11031-013-9355-4

[B22] Hofmann W. Van Dillen L. (2012). Desire: the new hot spot in self-control research. *Curr. Dir. Psychol. Sci.* 21 317–322. 10.1177/0963721412453587

[B23] Hofmann W. Wisneski D. C. Brandt M. J. Skitka L. J. (2014). Morality in everyday life. *Science* 345 1340–1343. 10.1126/science.1251560 25214626

[B24] Hynie M. MacDonald T. K. Marques S. (2006). Self-conscious emotions and self-regulation in the promotion of condom use. *Pers. Soc. Psychol. Bull.* 32 1072–1084. 10.1177/0146167206288060 16861311

[B25] Idson L. C. Liberman N. Higgins E. T. (2004). Imagining how you’d feel: the role of motivational experiences from regulatory fit. *Pers. Soc. Psychol. Bull.* 30 926–937. 10.1177/0146167204264334 15200698

[B26] Kahneman D. (2011). *Thinking, Fast and Slow.* New York, NY: Farrar, Straus and Giroux.

[B27] Katzir M. Eyal T. Meiran N. Kessler Y. (2010). Imagined positive emotions and inhibitory control: the differentiated effect of pride versus happiness. *J. Exp. Psychol.Learn. Mem. Cogn.* 36 1314–1320. 10.1037/a0020120 20804298

[B28] Klass E. T. (1990). “Guilt, shame, and embarrassment: Cognitive-behavioral approaches,” in *Handbook of Social and Evaluation Anxiety*, ed. Leitenberg H. (New York, NY: Plenum Press), 385–414. 10.1007/978-1-4899-2504-6_13

[B29] Kotabe H. P. Hofmann W. (2015). On integrating the components of self-control. *Perspect. Psychol. Sci.* 10 618–638. 10.1177/1745691615593382 26386000

[B30] Lewin K. (1951). *Field Theory in Social Science: Selected Theoretical Papers.* Oxford: Harpers.

[B31] Lewis H. B. (1971). Shame and guilt in neurosis. *Psychoanal. Rev.* 58 419–438. 5150685

[B32] Louro M. J. Pieters R. Zeelenberg M. (2007). Dynamics of multiple-goal pursuit. *J. Pers. Soc. Psychol.* 93 174–193. 10.1037/0022-3514.93.2.174 17645394

[B33] MacInnis D. J. Patrick V. M. (2006). A spotlight on affect: the role of affect and affective forecasting in self-regulation and impulse control. *J. Consum. Psychol.* 16 224–231. 10.1207/s15327663jcp1603_4

[B34] Mellers B. A. (2000). Choice and the relative pleasure of consequences. *Psychol. Bull.* 126 910–924. 10.1037/0033-2909.126.6.910 11107882

[B35] Mellers B. A. McGraw A. P. (2001). Anticipated emotions as guides to choice. *Curr. Dir. Psychol. Sci.* 10 210–214. 10.1111/1467-8721.00151

[B36] Metcalfe J. Mischel W. (1999). A hot/cool-system analysis of delay of gratification: dynamics of willpower. *Psychol. Rev.* 106 3–19. 10.1037//0033-295x.106.1.3 10197361

[B37] Mukhopadhyay A. Johar G. V. (2007). Tempted or not? The effect of recent purchase history on responses to affective advertising. *J. Consum. Res.* 33 445–453. 10.1086/510218

[B38] Ortony A. Turner T. J. (1990). What’s basic about basic emotions? *Psychol. Rev.* 97 315–331. 10.1037//0033-295x.97.3.315 1669960

[B39] Patrick V. M. Chun H. H. Macinnis D. J. (2009). Affective forecasting and self-control: why anticipating pride wins over anticipating shame in a self-regulation context. *J. Consum. Psychol.* 19 537–545. 10.1016/j.jcps.2009.05.006

[B40] Paulhus D. L. (1998). Interpersonal and intrapsychic adaptiveness of trait self-enhancement: a mixed blessing? *J. Pers. Soc. Psychol.* 74 1197–1208. 10.1037//0022-3514.74.5.1197 9599439

[B41] Perugini M. Bagozzi R. P. (2001). The role of desires and anticipated emotions in goal-directed behaviours: broadening and deepening the theory of planned behaviour. *Br. J. Soc. Psychol.* 40 79–98. 10.1348/014466601164704 11329835

[B42] Phillips P. A. Rolls B. J. Ledingham J. Forsling M. L. Morton J. J. Crowe M. J. (1984). Reduced thirst after water deprivation in healthy elderly men. *N. Engl. J. Med.* 311 753–759. 10.1056/nejm198409203111202 6472364

[B43] Richard R. Vries N. K. Pligt J. (1998). Anticipated regret and precautionary sexual behavior. *J. Appl. Soc. Psychol.* 28 1411–1428. 10.1111/j.1559-1816.1998.tb01684.x 17453577

[B44] Rook D. W. (1987). The buying impulse. *J. Consum. Res.* 14 189–199. 10.1086/209105

[B45] Rozin P. Royzman E. B. (2001). Negativity bias, negativity dominance, and contagion. *Pers. Soc. Psychol. Rev.* 5 296–320. 10.1207/s15327957pspr0504_2

[B46] Schachter S. Goldman R. Gordon A. (1968). Effects of fear, food deprivation, and obesity on eating. *J. Pers. Soc. Psychol.* 10 91–97. 10.1037/h0026284 5725907

[B47] Shefrin H. M. Thaler R. H. (1988). The behavioral life-cycle hypothesis. *Econ. Inq.* 26 609–643. 10.1111/j.1465-7295.1988.tb01520.x

[B48] Shimoni E. Asbe M. Eyal T. Berger A. (2016). Too proud to regulate: the differential effect of pride versus joy on children’s ability to delay gratification. *J. Exp. Child Psychol.* 141 275–282. 10.1016/j.jecp.2015.07.017 26319959

[B49] Shiv B. Huber J. (2000). The impact of anticipating satisfaction on consumer choice. *J. Consum. Res.* 27 202–216. 10.1086/314320

[B50] Simonson I. (1992). The influence of anticipating regret and responsibility on purchase decisions. *J. Consum. Res.* 19 105–118.

[B51] Strack F. Deutsch R. (2004). Reflective and impulsive determinants of social behavior. *Pers. Soc. Psychol. Rev.* 8 220–247. 10.1207/s15327957pspr0803_1 15454347

[B52] Summerville A. (2011). The rush of regret: a longitudinal analysis of naturalistic regrets. *Soc. Psychol. Pers. Sci.* 2 627–634. 10.1177/1948550611405072 9592630

[B53] Tangney J. P. (1999). “The self-conscious emotions: Shame, guilt, embarrassment and pride,” in *Handbook of Cognition and Emotion*, eds Dalgleish T. Power M. (West Sussex: Wiley), 541–568. 10.1002/0470013494.ch26

[B54] Tangney J. P. Dearing R. L. (2002). *Shame and Guilt.* New York, NY: Guilford.

[B55] Taylor G. (ed.) (1985). *Pride, Shame, and Guilt. Emotions of Self-Assessment.* Oxford: Clarendon.

[B56] Thaler R. H. Shefrin H. M. (1981). An economic theory of self-control. *J. Polit. Econ.* 89 392–406. 10.1086/260971

[B57] Thaler R. Sunstein C. (2008). *Nudge: Improving Decisions about Health, Wealth, and Happiness.* New Haven, CT: Yale University Press.

[B58] Tracy J. L. Robins R. W. (2004). Putting the self into self-conscious emotions: a theoretical model. *Psychol. Inq.* 15 103–125. 10.1207/s15327965pli1502_01

[B59] Tracy J. L. Robins R. W. (2007). The psychological structure of pride: a tale of two facets. *J. Pers. Soc. Psychol.* 92 506–525. 10.1037/0022-3514.92.3.506 17352606

[B60] Trope Y. Liberman N. (2010). Construal-level theory of psychological distance. *Psychol. Rev.* 117 440–463. 10.1037/a0018963 20438233 PMC3152826

[B61] Tversky A. Kahneman D. (1991). Loss aversion in riskless choice: a reference-dependent model. *Q. J. Econ.* 106 1039–1061. 10.2307/2937956

[B62] Wilson T. D. Gilbert D. T. (2003). Affective forecasting. *Adv. Exp. Soc. Psychol.* 35 345–411.

