# Supplementary material for: How Anticipated Emotions Guide Self-Control Judgments
Source: Front Psychol. 2019 Jul 23;10:1614. doi: 10.3389/fpsyg.2019.01614 (PMC6664080; doi:10.3389/fpsyg.2019.01614)
Supplement: Supplementary file 1 [file Data_Sheet_1.docx]

Methodological Details Appendix and Supplementary Materials for

**How Anticipated Emotions Guide Self-Control Judgments**

**This file includes:**

1. Supplementary Figure 1: Slider Scales
2. Supplementary Table 1: Descriptive Statistics in Study 2
3. Materials: Self-Control Scenarios Used
4. Supplementary Study 4: The Influence of Prior self-control exertion (including Supplementary Figure 2)
5. **Supplementary Figure 1: Slider Scales**

| 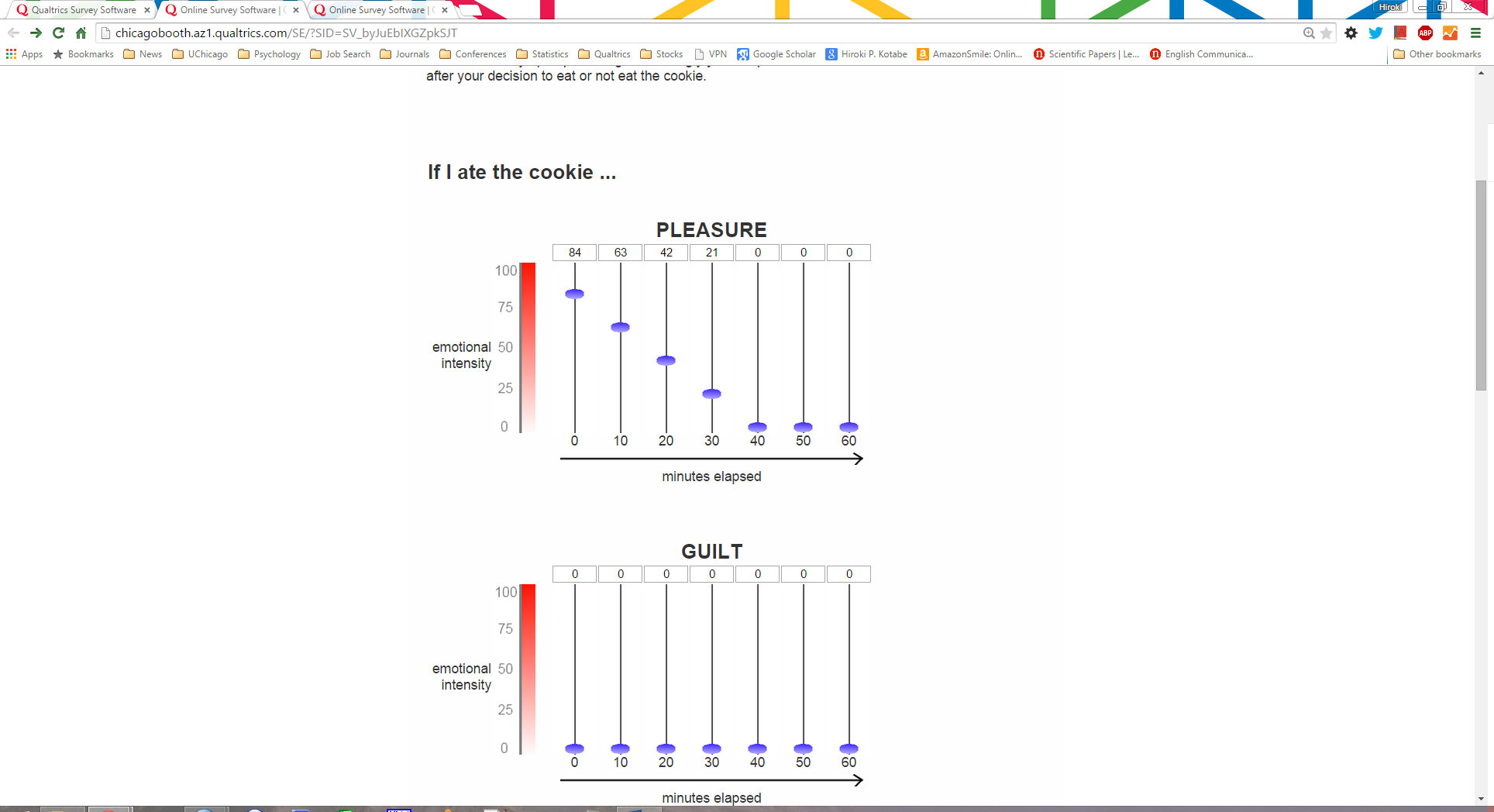 |
| --- |
| *Supplementary Figure 1.* A screenshot of one of the sets of slider scales we used in the AReA Events Task to capture the intensity and duration of anticipated emotions. |

1. **Supplementary Table 1**

Supplementary Table 1. *Descriptive Statistics for Pleasure, Guilt, Pride, and Frustration Area Measures Across Self-Control Scenarios in Study 2*

| Pleasure Area | | | | | |
| --- | --- | --- | --- | --- | --- |
|  |  | *Food* | *Alcohol* | *Sex* | *Aggression* |
| Mean |  | 319.38 | 302.31 | 313.09 | 243.76 |
| Std. Deviation |  | 162.18 | 171.90 | 206.64 | 200.41 |
| Minimum |  | 18 | 0 | 0 | 0 |
| Maximum |  | 700 | 648 | 700 | 700 |
| Percentiles | 25 | 197 | 167 | 125 | 71 |
|  | 50 | 290 | 290 | 330 | 213 |
|  | 75 | 412 | 428 | 496 | 380 |
| Guilt Area | | | | | |
|  |  | *Food* | *Alcohol* | *Sex* | *Aggression* |
| Mean |  | 422.41 | 446.33 | 541.03 | 348.71 |
| Std. Deviation |  | 184.41 | 187.93 | 187.98 | 227.56 |
| Minimum |  | 4 | 0 | 7 | 0 |
| Maximum |  | 700 | 700 | 700 | 700 |
| Percentiles | 25 | 285 | 300 | 438 | 157 |
|  | 50 | 427 | 470 | 608 | 325 |
|  | 75 | 574 | 599 | 700 | 572 |
| Pride Area | | | | | |
|  |  | *Food* | *Alcohol* | *Sex* | *Aggression* |
| Mean |  | 486.38 | 455.26 | 460.73 | 374.54 |
| Std. Deviation |  | 193.39 | 202.13 | 234.24 | 233.26 |
| Minimum |  | 0 | 0 | 0 | 0 |
| Maximum |  | 700 | 700 | 700 | 700 |
| Percentiles | 25 | 340 | 310 | 247 | 155 |
|  | 50 | 528 | 450 | 532 | 360 |
|  | 75 | 655 | 660 | 692 | 588 |
| Frustration Area | | | | | |
|  |  | *Food* | *Alcohol* | *Sex* | *Aggression* |
| Mean |  | 328.58 | 237.43 | 229.48 | 386.36 |
| Std. Deviation |  | 171.64 | 178.95 | 197.62 | 196.78 |
| Minimum |  | 0 | 0 | 0 | 0 |
| Maximum |  | 700 | 700 | 700 | 700 |
| Percentiles | 25 | 207 | 80 | 50 | 241 |
|  | 50 | 332 | 230 | 185 | 399 |
|  | 75 | 454 | 338 | 395 | 548 |

1. **Self-Control Scenarios**

**Food**
 
Please try to imagine the following situation as vividly as you can.
 
You’ve been dieting for a few weeks and have made great progress. You’ve lost a substantial amount of fat, and have put on some muscle in the parts of your body you were working on. You feel very good about yourself because you haven’t slipped from your diet or workout regimen once. With unhealthy foods, you’ve come close to losing control. You have a big appetite for certain unhealthy foods (e.g., moist cake, pizza, cheeseburgers, fried dumplings).

[please contact authors for images]

Now imagine that you showered at the gym and left directly for a dinner party at a friend’s house. When you arrive, you see that your friend has among other foods, the unhealthy food you find unbelievably delicious and mouth-watering. You can’t help but make your way directly in front of this delicious but unhealthy and fatty food.
 
You are faced with this dilemma. If you eat it, you will definitely enjoy it, but you will break your diet. If you don’t eat it, you will maintain your diet, but you won’t satiate that immense desire for it.
 

**Alcohol**
 
Please try to imagine the following situation as vividly as you can.
 
You are out for a night out downtown with your new coworkers. You find a nice nightclub to go dancing. It’s dark in the nightclub but you are still somewhat nervous about dancing in front of your new coworkers.
 
To break the ice, one of your coworkers buys a round of shots. Normally, you’d be excited to have a drink and loosen up for a night of fun dancing. But, this time it’s different because you know you have to drive more than 10 miles home, and you recently got a ticket for driving under the influence of alcohol.

[please contact authors for images]

You say, “no thanks”, to the shots, but your coworker insists, and gets you your favorite mix drink instead. They all look like they are having a good time, getting loose. The alcohol is tempting, but you know you will break your promise to yourself to not have any drinks before driving.
 
You are faced with this dilemma. If you take the drink from your coworker, you will surely loosen up and have more fun, but you would be breaking your promise to not drink before driving. If you don’t take the drink, you’ll keep your promise to yourself but you probably won’t have that much fun at the nightclub with your coworkers.

**Sex (if participant was male)**
 
Please try to imagine the following situation as vividly as you can (and please assume the other character's gender matches the gender you are sexually attracted to).
 
You’re out at your friend’s Halloween party. Your partner is out of town so you’re attending by yourself. You’ve had a few drinks and are having a really good time. Your friend hangs out with a different crowd from you so you’ve had a chance to meet a lot of new people.
 
There is a very attractive woman who you’ve struck a good conversation with. She is intelligent and stunningly beautiful, but you know you shouldn’t flirt with her because you have a partner who you are faithful to. Despite telling her about your partner, after talking and laughing together for a while, she gets very close to you and unexpectedly, she kisses you sensually.

[please contact authors for images]

You push her away gently and apologize, telling her that you’re taken. She says, “I won’t tell”. You don’t see your friend around and none of the people around you know you are in a relationship. You could kiss her back or not.
 
You are faced with this dilemma. The woman is very attracted to you. If you kiss her, you know she would take things farther, but you would be unfaithful to your partner. If you don’t kiss her, you will remain faithful to your partner, but you won’t spend any more time with this woman.
 

**Aggression (if participant was male)**
 
Please try to imagine the following situation as vividly as you can.
 
You’re driving your car and you reach a stop sign, so you stop. As you proceed, a car runs the stop sign on the perpendicular street and rams into the right side of your car. Your airbags don’t deploy, you’re shaken up, and you see that your car is wrecked.
 
You’re happy to not be hurt badly. You get out of your car to resolve the issue with this man. As you get out, you hear him screaming at you. He’s insulting you, calling you stupid and ignorant. It seems like he might be drunk.
 
You try to calm him down and resolve things maturely, but the man keeps insulting you. He says the whole thing is your fault but you know that is crazy. You put your hand on his shoulder to try to calm him, but he aggressively pushes you and *spits in your face*.

[please contact authors for images]

Now, you know that you try to always be nonviolent. You are a peaceful person, and it is very important for you to keep it cool in difficult situations. But this man is pushing you to your limits. He is much smaller than you and somewhat scrawny. You know that you could easily shut him up with one hard slap to his face.
 
You are faced with this dilemma. If you slap the man, you will shut him up, but you will break your principle of nonviolence. If you don’t slap him, you will stick with your nonviolence principle, but he will keep being hostile toward you.

**Sex (if participant was female)**
 
Please try to imagine the following situation as vividly as you can (and please assume the other character's gender matches the gender you are sexually attracted to).

You’re out at your friend’s Halloween party. Your partner is out of town so you’re attending by yourself. You’ve had a few drinks and are having a really good time. Your friend hangs out with a different crowd from you so you’ve had a chance to meet a lot of new people.
 
There is a very attractive man who you’ve struck a good conversation with. He is intelligent and stunningly handsome, but you know you shouldn’t flirt with him because you have a partner who you are faithful to. Despite telling him about your partner, after talking and laughing together for a while, he gets very close to you and unexpectedly, he kisses you sensually.

 
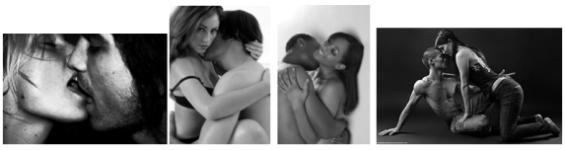


You push him away gently and apologize, telling him that you’re taken. He says, “I won’t tell”. You don’t see your friend around and none of the people around you know you are in a relationship. You could kiss him back or not.
 
You are faced with this dilemma. The man is very attracted to you. If you kiss him, you know he would take things farther, but you would be unfaithful to your partner. If you don’t kiss him, you will remain faithful to your partner, but you won’t spend any more time with this man.

**Aggression (if participant was female)**
 
Please try to imagine the following situation as vividly as you can.
 
You’re driving your car and you reach a stop sign, so you stop. As you proceed, a car runs the stop sign on the perpendicular street and rams into the right side of your car. Your airbags don’t deploy, you’re shaken up, and you see that your car is wrecked.

You’re happy to not be hurt badly. You get out of your car to resolve the issue with this woman. As you get out, you hear her screaming at you. She’s insulting you, calling you stupid and ignorant. It seems like she might be drunk.
 
You try to calm her down and resolve things maturely, but the woman keeps insulting you. She says the whole thing is your fault but you know that is crazy. You put your hand on her shoulder to try to calm her, but she aggressively pushes you and *spits in your face*.


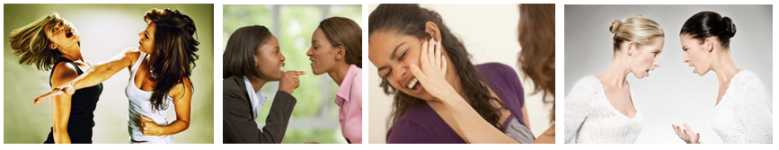


Now, you know that you try to always be nonviolent. You are a peaceful person, and it is very important for you to keep it cool in difficult situations. But this woman is pushing you to your limits. She is much smaller than you and somewhat scrawny. You know that you could easily shut her up with one hard slap to her face.
 
You are faced with this dilemma. If you slap the woman, you will shut her up, but you will break your principle of nonviolence. If you don’t slap her, you will stick with your nonviolence principle, but she will keep being hostile toward you.

1. **Supplementary Study 4: The Influence of Prior self-control exertion**

In Supplementary Study 4, we investigated the effects of *situating* this process under conditions of prior self-control exertion vs. no prior self-control exertion. Based on recent evidence causally linking prior self-control exertion to lower-level processing and increased reward-sensitivity (e.g., [Bruyneel & Dewitte, 2012](#_ENREF_11); [Vohs et al., 2013](#_ENREF_64); [Wagner et al., 2013](#_ENREF_65); [Wagner & Heatherton, 2013](#_ENREF_66); [Wan & Agrawal, 2011](#_ENREF_67)), we hypothesized that prior self-control exertion draws focus to anticipated pleasure and away from anticipated guilt, and this would be reflected in weighting of these anticipated emotions into self-control judgments.

**Method**

**Participants and design.** 181 US-based adults (86 men, 93 women) recruited on AMT participated in this two-group (prior self-control exertion vs. no prior self-control exertion) between-subjects experiment. Ages ranged from 18 to 68 (*M* = 33.57, *SD* = 12.26). Ethnicities were reported nonexclusively: 152 participants identified as White/Caucasian, 12 identified as Asian/Asian American, 10 identified as Black/African American, 6 identified as Hispanic/Latino, and 1 identified as Native American/Alaska Native.

**Procedure.** Participants were randomly assigned to the prior self-control exertion condition or a control condition. To manipulate prior self-control exertion, we used the attentional control task ([Baumeister et al., 1998](#_ENREF_7)). In this task, participants are asked to do a task purportedly about the “nonverbal assessments of others’ personalities.” The task involves watching a silent video clip, seven minutes long, of a woman being interviewed by an off-camera interviewer. This video clip is placed in the top-left of the screen. In addition, a smaller video clip, also with no audio, is placed on the bottom-right of the screen. This smaller video clip simply displays a series of common one-syllable words (e.g., “glue”) for 10 seconds each in plain black text against a plain white background. This second video clip is sized to be large enough to be easy to see but small enough to not dominate the screen. The words displayed in the small video clip are irrelevant to what is happening in the main video clip. Participants in the prior self-control exertion condition are told that for the task to work effectively, it is “very important that you try your hardest not to read or look at any words that may appear on the screen,” thus effectively getting them to direct their attention for an extended period. Research suggests that directed attention fatigue impairs self-control and executive functioning (Kaplan & Berman, 2010). In the control condition, participants are not given any instructions regarding the irrelevant words and thus are free to attend to the video naturally (i.e., without directed attention).

Following the experimental manipulation, we used the same procedure and scales as in Study 2, but out of concern that influence of the experimental manipulation might not last through four temptation scenarios, we randomly presenting two of the four temptation scenarios to each participant. Thus, all four scenarios were still presented across participants, but each participant received only two. Therefore, we effectively shortened the task but still sampled widely from prototypical self-control scenarios as in Study 2.

**Data Analytic Procedures**

The data analytic procedures were the same as those used to analyze Study 2 except that we entered a dummy-coded prior-self-control variable and its two-way interactions with scenario into the main effects and weighting models and the former into the trajectory models.

**Results and Discussion**

**Main effects.** The experimental manipulation of prior self-control exertion did not have significant main effects on the predicted emotion areas nor on self-control judgments. We do not interpret these null findings, but note that manipulations of prior self-control exertion have small and complex effects that are not yet well-understood or agreed upon. Furthermore, to our knowledge, there is no research on the effects of prior self-control exertion of the sort of self-control judgments we measured in our study.

**Trajectories.** In the control group, we again found that participants anticipated that pleasure and frustration would decay, *B* = -7.71, *p* < .001, 95% CI [-8.37, -7.05] and *B* = -5.37, *p* < .001, 95% CI [-6.01, -4.72], respectively, faster than pride and guilt, *B* = 1.98, *p* < .001, 95% CI [1.37, 2.59] and *B* = 2.24, *p* < .001, 95% CI [1.65, 2.82], respectively, lending additional support to H2. The latter self-conscious emotions were again anticipated to intensify. Moderated regression analysis using a dummy-coded variable for self-conscious emotions confirmed that the difference in slopes between anticipated primitive emotions and anticipated self-conscious emotions was again significant, *B* = -11.11, *p* < .001, 95% CI [-13.79, -8.43].

Comparatively, participants in the prior self-control exertion condition anticipated that pleasure, *B* = -6.82, *p* < .001, 95% CI [-7.50, -6.19], and frustration, *B* = -4.55, *p* < .001, 95% CI [-5.20, -3.91], would decay slower, though these differences were marginal, *B* = 0.87, *p* = .068, 95% CI [-0.07, 1.80] and *B* = 0.81, *p* = .081, 95% CI [-0.10, 1.73]. Prior self-control exertion had no effects on the trajectories of anticipated pride and guilt. Moderated regression using a dummy-coded variable for self-conscious emotions confirmed that the difference in slopes between the anticipated primitive emotions and the anticipated self-conscious emotions was still significant, *B* = -9.24, *p* < .001, 95% CI [-11.91, -6.57]. These results provide weak evidence that prior self-control exertion results in the anticipation that pleasure and frustration will last longer in self-control scenarios.

**Weighting.** We tested whether prior self-control exertion may affect the weighting of anticipated pleasure and guilt into self-control judgments. To do so, we regressed self-control judgments on the anticipated emotion variables and their interactions with the manipulation of prior self-control exertion. In the control group, anticipated pleasure, β = -0.12, *p* = .052, 95% CI [-0.25, 0.00], and anticipated frustration, β = -0.16, *p* = .015, 95% CI [-0.29, -0.03], marginally to significantly predicted judgments favoring less self-control whereas anticipated guilt, β = 0.52, *p* < .001, 95% CI [0.38, 0.66], predicted judgments favoring more self-control (anticipated pride was not a significant predictor this time, β = 0.03, *p* = .684, 95% CI [-0.11, 0.16]). The significant results here are consistent with H1a and H1b. We also replicated the result that anticipated guilt is weighted most—contrasts comparing absolute beta coefficients indicated that the anticipated guilt effect was significantly larger than the average effect of the other three anticipated emotions, β_dif_ = 0.42, *p* < .001, 95% CI [0.24, 0.60], and also significantly larger than the individual effects of anticipated pleasure, β_dif_ = 0.40, *p* < .001, 95% CI [0.20, 0.60], anticipated pride, β_dif_ = 0.49, *p* < .001, 95% CI [0.25, 0.74], and anticipated frustration, β_dif_ = 0.36, *p* < .001, 95% CI [0.18, 0.55], supporting H3a. As for the relatively weak weighting of anticipated pride, the anticipated pride effect was significantly smaller than the average effect of the other anticipated emotions, β_dif_ = -0.24, *p* = .005, 95% CI [-0.41, -0.07], and also significantly smaller than the individual effect of anticipated guilt (see above), but not the individual effects of anticipated pleasure, β_dif_ = -0.09, *p* = .286, 95% CI [-0.27, 0.08], and anticipated frustration, β_dif_ = -0.13, *p* = .168, 95% CI [-0.32, 0.06], supporting H3b, albeit, with weaker evidence than in Study 2.

***Prior self-control exertion shifts weight to anticipated pleasure.*** As shown in Table 2 (main article), the manipulation of prior self-control exertion had multiple significant interactive effects on the weighting of anticipated emotions. Prior self-control exertion particularly increased how much people’s self-control judgments were guided by anticipated pleasure (see Supplementary Figure 2, left panel). In the prior self-control exertion group, pleasure was the strongest predictor of self-control judgments, β = -0.37, *p* < .001, 95% CI [-0.51, -0.24], and the difference between conditions was statistically significant, β = 0.25, *p* = .007, 95% CI [-0.07, 0.43]. Contrasts comparing absolute beta coefficients indicated that the anticipated pleasure effect was larger than the average effect of the other three anticipated emotions, β_dif_ = 0.19, *p* = .032, 95% CI [0.02, 0.36], and also marginally to significantly larger than the individual effects of anticipated guilt, β_dif_ = 0.20, *p* = .068, 95% CI [-0.02, 0.42], and anticipated pride, β_dif_ = 0.18, *p* = .041, 95% CI [0.01, 0.35], but not anticipated frustration, β_dif_ = 0.18, *p* = .130, 95% CI [-0.05, 0.42].

*Supplementary Figure 2.* Moderating role of prior self-control exertion in Supplementary Study 4: Prior self-control exertion increased how much participants weighted anticipated pleasure (left panel) and decreased how much participants weighted anticipated guilt (right panel) into self-control judgments.

***… and away from anticipated guilt.*** At the same time, prior self-control exertion evidently decreased the degree to which people’s self-control judgments were guided by anticipated guilt, supporting H4a (see Supplementary Figure 2, right panel). In the group who underwent prior-self-control exertion, anticipated guilt was no longer the strongest predictor, β = -0.16, *p* = .013, 95% CI [0.04, 0.31], and the decrease in weighting of anticipated guilt as compared with the control group was statistically significant, β = -0.35, *p* < .001, 95% CI [0.16, 0.54].

**Pride focus?** Unexpectedly, the manipulation of prior self-control exertion also had a marginal effect on the weighting of anticipated pride (see Table 2, main article), which may explain the absence of a significant effect of prior self-control exertion on self-control judgments. Anticipated pride was not a significant predictor of self-control judgments in the control group but it was a statistically significant predictor in the prior self-control exertion group, β = 0.20, *p* = .004, 95% CI [0.06, 0.33], and the group-level difference was marginally significant, β = 0.17, *p* = .080 95% CI [-0.02, 0.36]. This result is consistent with the proposal that pride as a secondary reward can substitute for pleasure as a foregone primary reward ([Tangney, Stuewig, & Mashek, 2007](#_ENREF_59)). We explored this possibility by conducting a three-way interaction between condition, anticipated pleasure, and anticipated pride on self-control judgments. The result was marginally significant, β = 0.10, *p* = .071, 95% CI [-0.01, 0.21], providing weak evidence that, in the prior self-control exertion group, weighting of anticipated pleasure decreased as weighting of anticipated pride increased—an indication of reward substitution. However, because these results were only marginally significant, these results should be interpreted with caution and suggest an interesting direction for future research.
